# Prof. Gibson and Mr. Crawford the Sculptor

**Published:** 1857-09

**Authors:** 


*Prof. Gibson and Mr. Crawford, the Sculptor.*

In the August number of the *Medical News and Library* we find a long letter from Prof. Gibson, detailing the history of his connection with the case of Mr. Crawford, the sculptor. We regret that Prof. Gibson has felt it necessary to make this defence, but all professional men will certainly regard it as full and satisfactory. It is probably known to most of our readers that Mr. Crawford, one of our distinguished American sculptors, has been laboring for a year past with a tumor in the orbit of the eye, which the ablest surgeons of Europe pronounce *malignant* in its character. Prof. Gibson, late of the University of Pennsylvania, being on a visit to Rome last winter, was requested by Dr. Smyth, the attending physician of Mr. Crawford, to examine the case and give the benefit of his advice. Before doing so Prof. Gibson explained that he had withdrawn from professional duties entirely and "did not wish to engage in any case whatever"—but as a friend and countryman he was willing to attend with Dr. Smyth, and while he remained in Rome to do everything in his power to serve Mr. Crawford, but that his services must be gratuitous. Says Dr. Gibson in his letter—"after minute examination and inquiry into the nature of the case, I remarked to Dr. Smyth—'it is evident that the eye, perfectly sound in itself, but pushed forward beyond the walls of the orbit more than half an inch, is acted upon by *pressure* from behind from a fluid or solid—that if from the former, an *exploring needle* might by discharging it cause the eye to resume its natural position, and be followed by a cure—that if on the contrary, *solid*, and, particularly, if of *malignant* character, no essential benefit can result from any treatment whatever, and that the case must, *necessarily*, in a few months terminate *fatally*.' Having obtained Mr. Crawford's consent to the use of the *exploring needle*, and being requested by Dr. Smyth to perform the operation, I engaged in it the next day, with every precaution and with the utmost delicacy, and in a few minutes was able to ascertain, with the utmost accuracy, that there was *no fluid*, but a *solid* tumor, which not only filled up the posterior part of the orbit, but might possibly have its origin in the brain."

As Prof. Gibson had anticipated and foretold, the tumor continued to develop rapidly—after which Mr. Crawford was sent to Paris, and received the attentions of the most eminent men there. Sometime in April, however, Mrs. Crawford wrote a letter to her friends in this country, which was published in the New York *Evening Post*, and wherein she expressly attributes the aggravation of Mr. Crawford's condition to the exploring operation by Prof. Gibson. All the circumstances of the case, certainly, render the letter of Mrs. Crawford, to say the least, very thoughtless and uncalled for. It is to this charge that Prof. Gibson especially designs to reply in the letter before us. This is a form of infliction to which the profession is much too frequently subjected—a family receives the benefit of the most careful, most reliable, most painfully matured advice and professional attentions—and yet if despite all the resources of our art the case proves unfortunate, the friends allow themselves to make disparaging remarks of the attendants, or, as in this case, assume to set up a non-professional opinion, in a case too where they cannot properly have any claim to capacity for giving an opinion of any worth, yet, we say, setting up this non-professional opinion to blast, so far as it may, the life-wrought reputation of the conscientious, skilful medical adviser. This evil is grossly unjust to the medical man, and eventually must in a great measure re-act unhappily to the interests of the community. Prof. Gibson has fortified himself with the opinions of Velpeau, Nelaton, Desmarres and Sichel, all confirming the views of Gibson in his diagnosis of the case, and heartily approving his operation with the *exploring needle* as every way proper in this case, and one of daily employment in doubtful cases of this character.—*Cin. Med. Observer.*

